# Effects of the electrical conductivity and orientation of silicon substrate on the synthesis of multi-walled carbon nanotubes by thermal chemical vapor deposition

**DOI:** 10.1186/1556-276X-8-110

**Published:** 2013-02-27

**Authors:** Hyonkwang Choi, Jaeseok Gong, Yeongjin Lim, Ki Hong Im, Minhyon Jeon

**Affiliations:** 1 Department of Nano Systems Engineering, Center for Nano Manufacturing, Inje University, Gimhae, Gyungnam 621-749, Republic of Korea; 2 Samsung Electronics Co, Suwon, Gyeonggi 443-742, Republic of Korea

**Keywords:** Multi-walled carbon nanotube, Catalytic nanoparticle, Substrate effect

## Abstract

We studied the effects of the electrical conductivity and orientation of silicon substrate on both catalytic Fe thin film and the structure and morphology of multi-walled carbon nanotube (MWNT) grown by low-pressure chemical vapor deposition. Both p-type Si(100) and Si(111) substrates with three different doping concentrations (high, low, undoped) were used to evaluate the formation of catalytic nanoparticles and the growth of MWNTs. The morphology of catalytic nanoparticles such as size and density was characterized by field-emission scanning electron microscopy, Cs-corrected energy-filtered transmission electron microscopy, and X-ray photoelectron spectroscopy. Structural characteristics of MWNTs grown on different combinations of silicon substrate orientation and electrical conductivities (*σ*) were also systematically analyzed. Based on the experimental results, growth modes of MWNTs could be controlled by choosing an appropriate combination of *σ* and orientation of Si substrates.

## Background

A large number of experimental parameters for multi-walled carbon nanotubes (MWNTs) grown by chemical vapor deposition (CVD) have been investigated including the type of thickness of catalytic metal films [1,2], the substrate temperature [3,4], the ammonia gas flow rates [5,6], and supporting substrate, etc. [7,8]. Among those parameters, the control of the catalyst particles is one of the most important factors that determine the structure and morphology of MWNT properties such as lengths, diameters, and density [9-11]. However, a basic growth mechanism explaining the way metallic atoms interact with carbon to nucleate, grow, and heal carbon nanotubes (CNTs) still needs to be understood. Previously, we investigated the effect of the electrical conductivity of the Si(100) substrate on the control of the growth of MWNTs and found that as the electrical conductivity of the silicon substrate increased, the average diameter of the MWNTs also increased while the density of MWNTs decreased [12]. Accordingly, the electrical conductivity (*σ*) of the substrate can be treated as a parameter for controlling the growth of MWNTs, which is another important parameter related to crystallographic orientation of the exposed substrate surface. Different orientations of silicon substrate play a role in CNT growth resulting from different surface energies. In this study, we report the effects of *σ* and orientation of the silicon substrate on the growth of MWNTs by thermal CVD. We also describe the role of proposed parameters that govern their growth kinetics and the knowledge about these.

## Methods

The p-type silicon substrates with different orientations and doping concentrations were prepared. The electrical characteristics for both Si(100) and Si(111) substrates at room temperature were measured using Hall measurement equipment (Ecopia HMS*-*3000, Bridge Technology, Chandler Heights, AZ, USA) and are summarized in Table 1. Silicon oxide layers on the substrate surfaces were removed using a conventional process with a buffered oxide etching solution. A 6-nm-thick iron film was deposited on the silicon substrate using an ion sputter. The CVD chamber was on standby and pumped down to a low pressure of less than 20 mTorr [13].

**Table 1:** Results of the Hall measurement by van der Pauw method 1 cm × 1 cm size

	**Bulk concentration**	**Conductivity**	**Mobility**
	**(/cm**^**3**^**)**	**(/Ω cm)**	**(Vs/cm)**
Si(100)			
U(100)	2.7 × 10^12^	6.7 × 10^-4^	15,000
L(100)	1.8 × 10^15^	9.8 × 10^-2^	350
H(100)	6.0 × 10^19^	4.3 × 10^2^	45
Si(111)			
U(111)	1.0 × 10^12^	1.7 × 10^-4^	59
L(111)	1.0 × 10^15^	6.1 × 10^-2^	370
H(111)	3.4 × 10^19^	8.9 × 10^2^	1,600

U, undoped; L, low; H, high.

Argon (Ar) gas was flowed into the chamber at a flow rate of 1,000 sccm in this experiment [14]. At the same time, while ammonia (NH_3_) gas with a flow rate of 140 sccm was flowed into the reactor, the substrates were heated up to the growth temperature of 900°C for 30 min and then maintained at 900°C for 5 min. Acetylene (C_2_H_2_) gas was supplied to synthesize MWNTs with a flow rate of 20 sccm for 10 min at 900°C [15,16]. After the growth of MWNTs, the chamber was cooled down to room temperature and purged with Ar ambient. This work has focused on the size contribution and formation of catalyst particles by supporting substrate orientation and conductivity. However, the samples must be taken to the instrument for *ex situ* analysis. Therefore, we have endeavored that the exposure of samples to air and moisture was minimized. Once the samples were taken out from the chamber and cooled off to room temperature, each sample was divided into small pieces for the characterization by field-emission scanning electron microscopy (FE-SEM; Hitachi S-4300SE, Hitachi, Ltd., Chiyoda-ku, Japan), Cs-corrected energy-filtered transmission electron microscopy (JEM-2200FS, JEOL Ltd., Akishima-shi, Japan), and X-ray photoelectron spectroscopy (XPS; AXIS Nova, Kratos Analytical Ltd., Manchester, UK). The XPS analysis was carried out using an Al K (1,486.6 eV) X-ray (*hν* = 1,486.6 eV) photoelectron spectrometer. The base pressure of the XPS system was 5.2 × 10^-9^ Torr.

## Results and discussion

**Figure 1:**
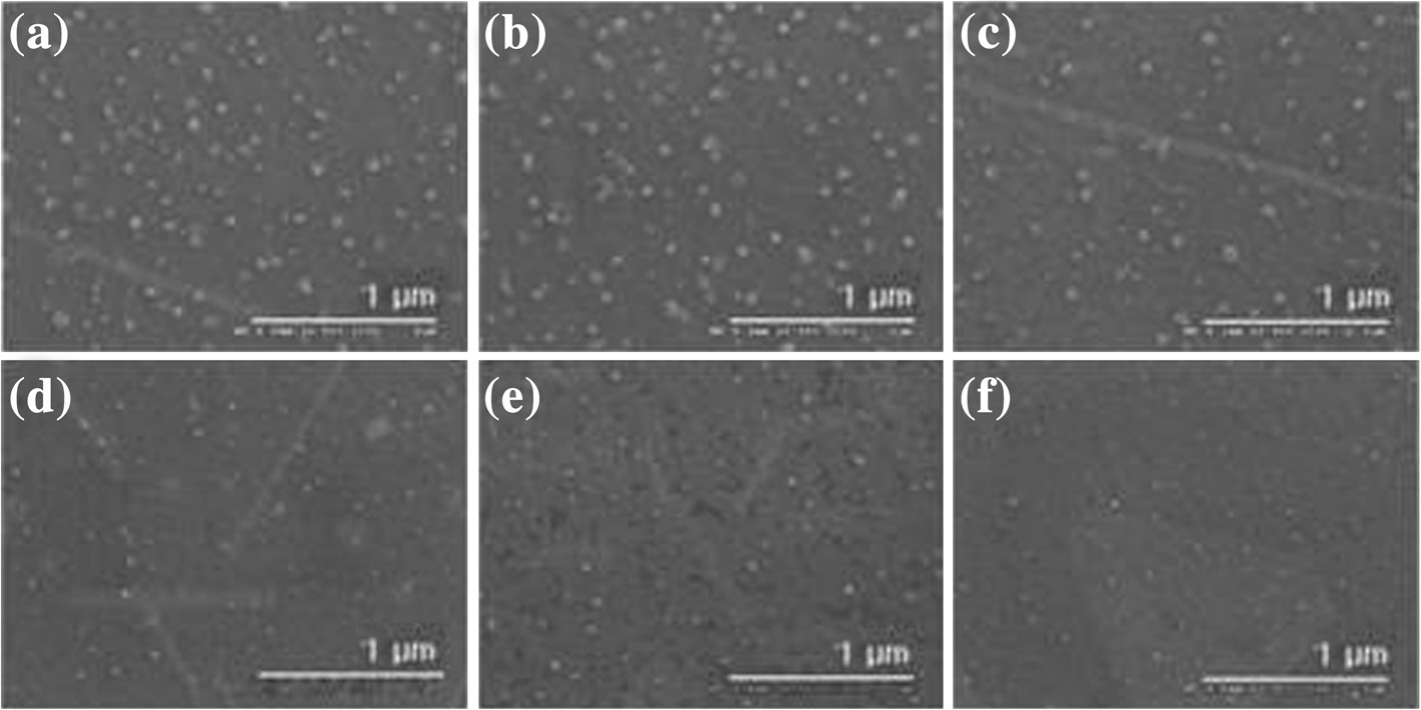
**Surface morphology of the samples.** (**a**) U(100), (**b**) L(100), (**c**) H(100), (**d**) U(111), (**e**) L(111), (**f**) H(111).

**Figure 2:**
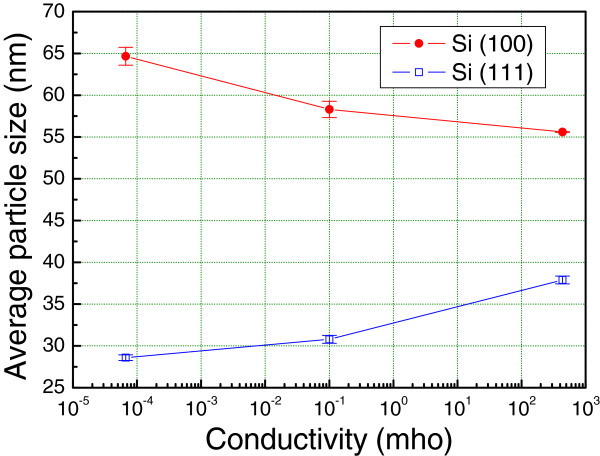
Plot of Fe particle average size and density versus Si(100) and Si(111) substrate electrical conductivity.

**Figure 3:**
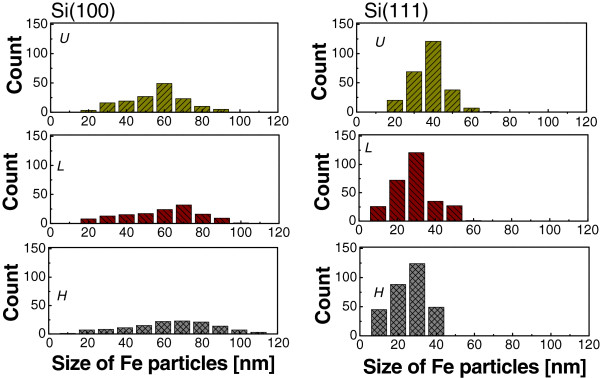
Histograms of the particle size distribution of Si(100) and Si(111) substrates.

Figure 1a,b,c,d,e,f illustrates the SEM images of Fe nanoparticles on Si(100) and Si(111) substrates at 900°C by applying the thermal chemical vapor deposition method. In the case of Si(100) substrate, as the *σ* of the silicon substrate increases, the average size of the Fe particles increases while the average density of the Fe particles decreases, as shown in Figure 1a,b,c. Figure 2 shows a plot of the average size of Fe particles versus the electrical conductivity of the Si(100) substrate. We conducted three different experiments and calculated the average values of the sizes and the densities of the nanoparticles to confirm the reproducibility of our experiment. We found that the average sizes of the Fe particles for substrates U(100), L(100), and H(100) were 55.6, 58.3, and 65.7 nm, respectively. This tendency is coincident with our previous results [9]. However, on the other hand, the average Fe particle size decreased as the electrical conductivity (*σ*) of Si(111) increased (Figure 1d,e,f). In the case of Si(111) substrate, as the *σ* of the silicon substrate increases, the average size of the Fe particles decreases while the average density of the Fe particles increases. It was found that the average sizes of the Fe particles for substrates U(111), L(111), and H(111) were 37.9, 30.8, and 28.6 nm, respectively. This result is opposite to that of the Si(100) substrate. Figure 3 shows the histograms of the particle size distribution on both Si(100) and Si(111) substrates.

The contrary tendency of Fe particle size according to substrate orientation could be explained that agglomeration and segregation of Fe particles were affected by atomic density, surface energy, and thermal conductivity of different Si surface orientations at the same thermal condition. The binding energy between Fe film and Si(100) substrate is smaller than that between Fe film and Si(111) substrate. In addition, the surface energy of Si(100), 2.13 J/cm^2^, is almost twice higher than that of Si(111), 1.23 J/cm^2^. Accordingly, it is expected that the catalytic particles could more easily migrate on Si(100) surface by thermal energy. Under these conditions, there exists a high probability of Fe particle agglomeration. Indeed, it was observed that the average diameter of Fe particles on Si(100) substrate was larger than that on Si(111) substrate. When the metal thin film is annealed, particles are formed by film coarsening, and then, they could agglomerate or break down through surface migration, driven by a thermally activated process resulting in a minimization of the surface energy of the metal film-substrate system.

**Figure 4:**
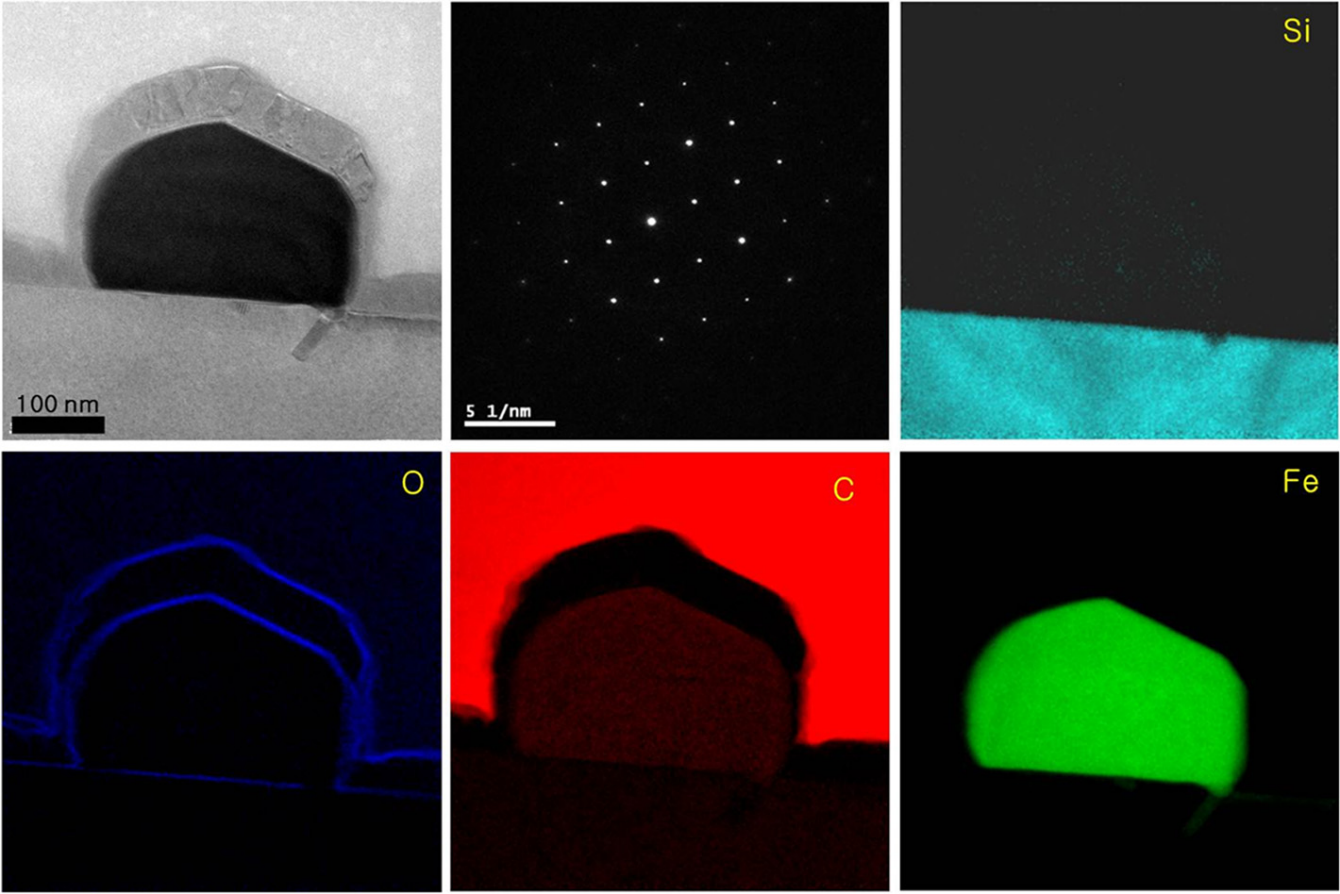
**Bright-field HR-TEM images and EELS elemental maps.** Showing the distribution of silicon (Si), oxygen (O), carbon (C), and iron (Fe) in plan views after introducing C_2_H_2_ at 900°C on silicon substrate U.

Zero-loss images and electron energy loss spectroscopy (EELS) elemental maps were examined to identify the distribution of Fe, O, and C on substrates U and H after introducing hydrocarbon gas for 5 s, as shown in Figure 4. After heat treatment, Fe particles were formed and oxidized. Oxygen might be provided from oxides on the Fe film after deposition on the silicon substrate or from residual natural oxides on the silicon surface. We found that the Fe particles on substrate U exhibited an oxygen layer, around 3 nm thick, on the surface of small Fe particles. In addition, a few layers of graphite were formed on the oxide layer of the oxidized Fe particle as in Figure 4. On the other hand, a certain amount of oxygen was present throughout the entire image at a very low intensity, and the graphite layers on substrate H were synthesized thicker than those on substrate U.

**Figure 5:**
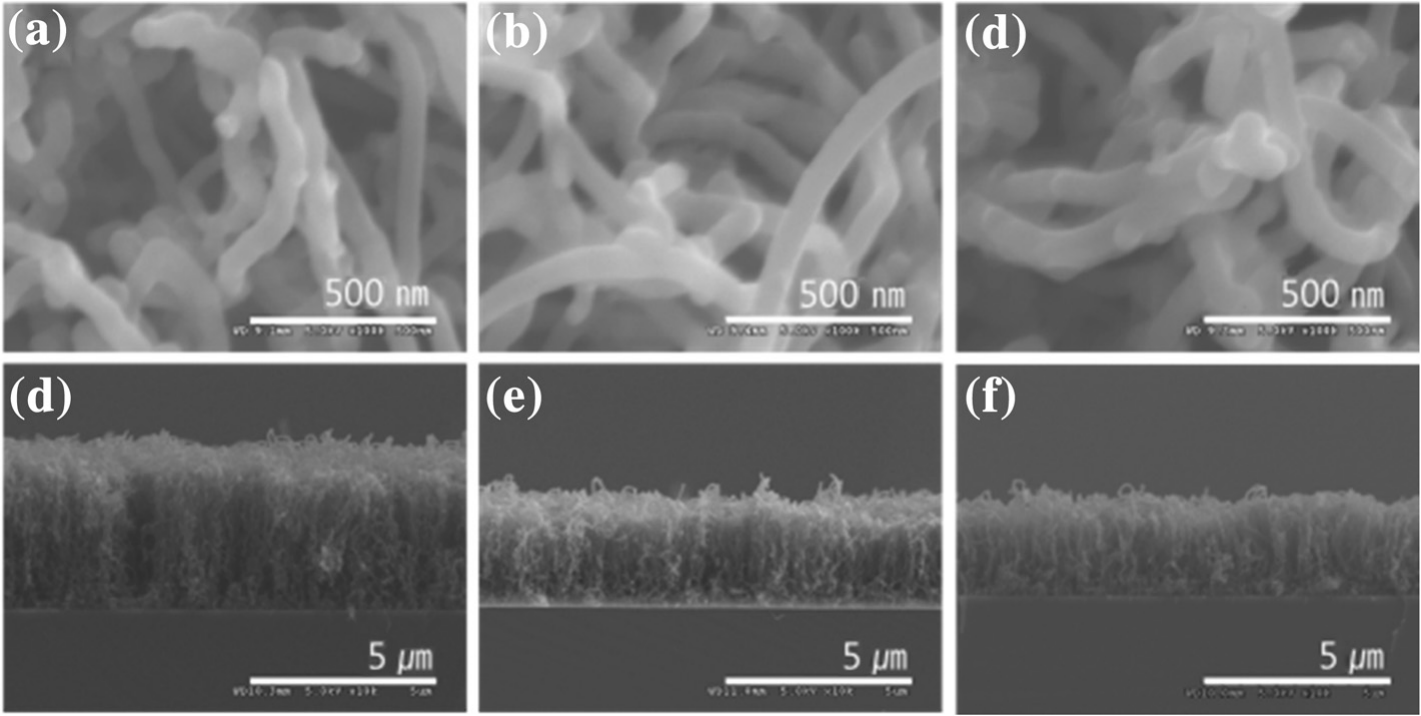
**FE-SEM micrographs of MWNTs grown on substrates U(100), L(100), and H(100).** (**a** to **c**) Plan view and (**d** to **f**) cross-sectional view.

**Figure 6:**
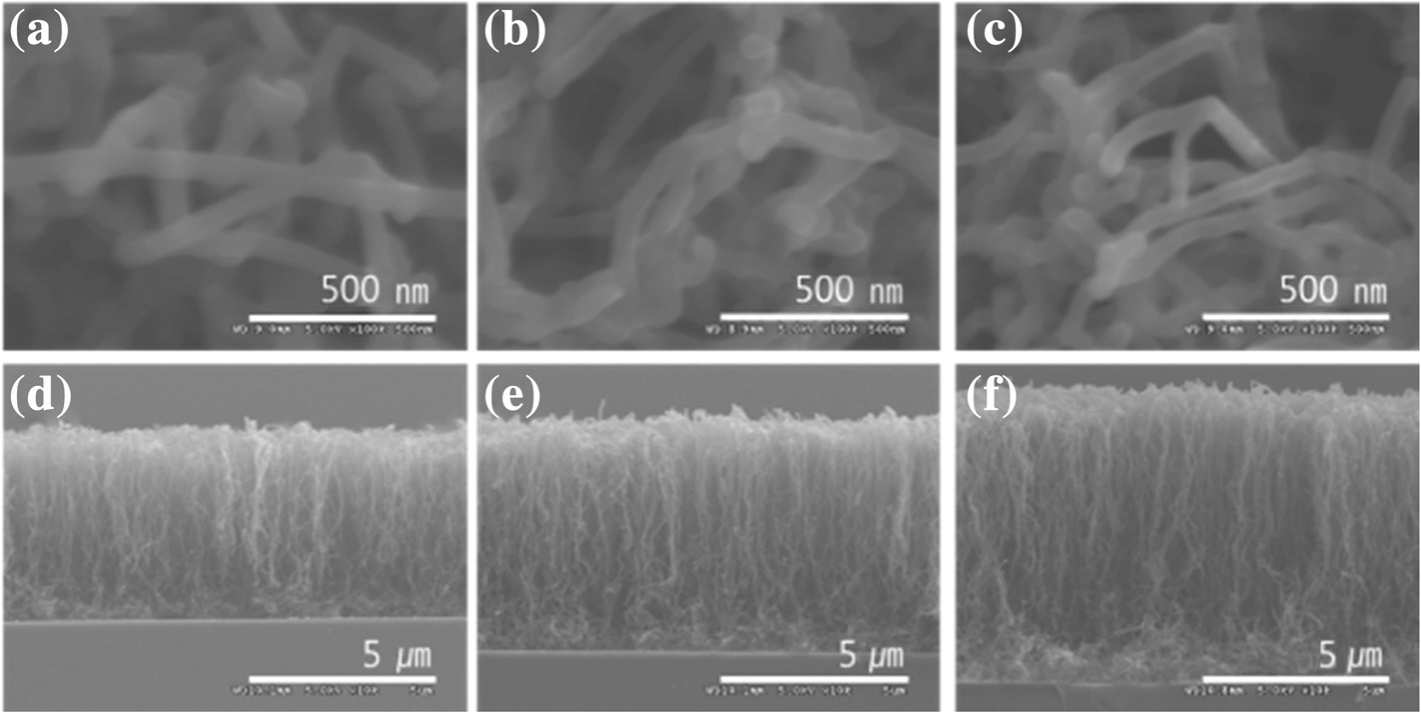
**FE-SEM micrographs of MWNTs grown on substrates U(111), L(111), and H(111).** (**a** to **c**) Plan view and (**d** to **f**) cross-sectional view.

**Figure 7:**
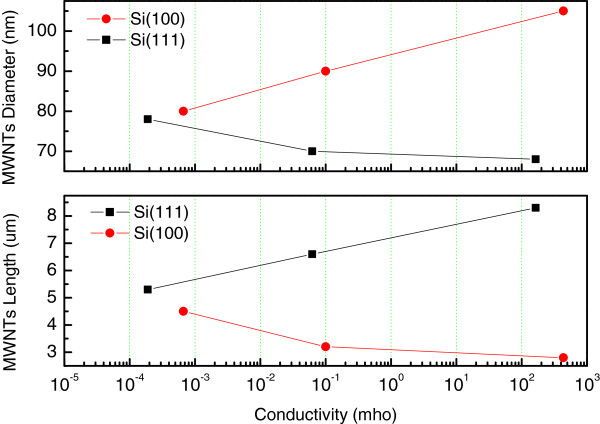
Plot of MWNT length and diameter versus electrical conductivity of the Si(100) and Si(111) substrates.

Figure 5a,b,c shows FE-SEM images of MWNTs grown on silicon substrates U(100), L(100), and H(100). Typical vertical-aligned MWNTs were grown on Si(100) substrates. In the case of Si(100) substrate, substrate U(100) with the lowest electrical conductivity has a dense distribution of thin and long MWNTs with average diameters of 30 to 40 nm and a length of around 25 μm. MWNTs with average diameters of 65 to 80 nm and a length of 5 to 6 μm were grown on substrate L(100), and thick and short MWNTs were grown on substrate H(100), which possessed the highest electrical conductivity. In this case, the average diameter and lengths of the MWNTS were found to be around 100 nm and 2 to 3 μm, respectively. For Si(111) substrate, however, the thin and long MWNTs were grown on H(111) substrate, while thick and short MWNTs were grown on substrate U(111), which possessed the lowest electrical conductivity compared with those of H(111) and L(111) substrates. Figure 6 shows cross-sectional and plan-view images of MWNTs grown on silicon substrates U(111), L(111), and H(111). Figure 7 shows a plot of length and diameter of MWNTs versus electrical conductivity of the Si(100) and Si(111) substrates. The average vertical lengths of MWNTs grown on U(111), L(111), and H(111) substrates are 5.3, 6.6, and 8.3 μm, respectively. On the other hand, the average diameter of MWNTs grown on U(111), L(111), and H(111) substrates are 78, 70, and 68 nm, respectively.

Generally, the diameter and length of carbon nanotubes were affected by catalytic metal particle sizes in the early stage of growth. Since the average Fe particle size on Si(100) substrate is larger than that on Si(111) substrate, MWNTs grown on Si(100) have larger diameter and shorter length than those grown on Si(111) substrate. As the electrical conductivity of Si(100) substrate increased, Fe particle size is increased, so carbon nanotubes with a short length and large diameter were grown. However, on the other hand, in the case of Si(111) substrate, as the electrical conductivity increased, smaller Fe particles were formed. Accordingly, MWNTs with small-diameter and long carbon nanotubes were synthesized.

## Conclusions

In this study, we report the effects of the orientation and electrical conductivity of silicon substrates on the synthesis of MWNTs by thermal CVD. It was found that the size and distribution of Fe particles on silicon substrate could be controlled by varying both orientation and *σ*. Accordingly, it is possible that the growth of MWNTs by thermal CVD could be also controlled by using the orientation and *σ*. In the case of Si(100) orientation, it was found that as the electrical conductivity of Si(100) substrates increased, the vertical growth of MWNTs was restrained while the radial growth was enhanced. On the other hand, in the case of Si(111) orientation, the situation is reversed. In this case, it was found that as the electrical conductivity of Si(111) substrates increased, the vertical growth of MWNTs was enhanced while the radial growth was restrained. More detailed investigation on this matter is in progress.

As a result, a strong correlation exists between the growth modes of the MWNTs and the combination of *σ* and orientation of the silicon substrate. Our results suggest that the combination of *σ* and orientation of the silicon substrate can be considered as an important parameter for controlling the growth modes of CNTs fabricated by thermal CVD, without the need to alter other growth parameters.

## Competing interests

The authors declare that they have no competing interests.

## Authors’ contributions

HC developed the conceptual framework and wrote the paper. JG and YL did the growth and characterization of the CNT. KHI helped in the experimental study and advised on the project. MJ supervised the work. All authors read and approved of the final manuscript.
